# Decomposing gender gaps in HIV service outcomes

**DOI:** 10.1136/bmjgh-2025-020900

**Published:** 2026-01-16

**Authors:** Gary Gaumer, Collins Gaba, Elad Daniels, Deborah Valerie Stenoien, Monica Jordan, VS Senthil Kumar, William Crown, Moaven Razavi, Allyala Nandakumar

**Affiliations:** 1 Institute for Global Health and Development, Brandeis University Heller School for Social Policy and Management, Waltham, Massachusetts, USA; 2 Lurie Institute for Disability Policy, Brandeis University Heller School for Social Policy and Management, Waltham, Massachusetts, USA

**Keywords:** Global Health, AIDS, Health services research, Delivery of Health Care, Health policy

## Abstract

This study uses Population-based HIV Impact Assessments survey data to examine factors associated with gender disparities in HIV outcomes. The analysis examined the share of adult males and females living with HIV who are aware of their status, are on treatment and have achieved viral load suppression across 13 African countries. The study then used the Blinder-Oaxaca statistical method to decompose these gaps into three core elements: (1) the part caused by observed differences in characteristics between the two groups, (2) the part caused by unobservable differences between the groups, often attributed to structural barriers and (3) the unexplained portion of the gap. The study then compares how these gaps and decompositions have changed over time. The model confirms that males have poorer outcomes than females across all three indicators. Factors contributing to these gender disparities include individual-level characteristics such as age, education and wealth, as well as structural barriers such as stigma, restrictive gender norms and lower health service utilisation among men. Although males generally possess more protective individual-level characteristics, these structural barriers offset their advantages, resulting in poorer outcomes across all indicators. The gap in service outcomes between men and women has decreased over time, with structural or cultural barriers showing the greatest improvement. Additional investment in and evaluation of male-friendly services is essential to understand what interventions have contributed to decreasing this gap. This knowledge should be used to inform future investments to support individual-level treatment outcomes and prevent new infections.

**WHAT IS ALREADY KNOWN ON THIS TOPIC**

- In President’s Emergency Plan for AIDS Relief-supported countries, there is a lower share of men living with HIV who are aware of their status, on treatment and have achieved viral load suppression when compared with women.
- Lower treatment coverage among men leads to worse individual health outcomes and is linked to increased infections among adolescent girls and young women.

**WHAT THIS STUDY ADDS**

- While there is significant evidence of these gaps, there are few studies that explore the factors that lead to this gap.
- The present analysis decomposes outcome gaps into three core components, including group-level differences in individual characteristics, such as wealth and education, structural components and other unobserved factors.
- The present study shows that structural factors account for a majority of the gap between men and women, and that structural barriers have decreased over time.

**HOW THIS STUDY MIGHT AFFECT RESEARCH, PRACTICE OR POLICY**

- Using these findings, programme managers and policy makers can invest in HIV services that mitigate structural barriers to care, such as cultural norms, stigma or other barriers to care for men.
- Current interventions targeting men have likely contributed to improved outcomes, yet future research is needed to evaluate these programmes in order to understand best practices and expand these interventions.

## Introduction

 Financial and infrastructure investments from country governments and international donors have allowed for widespread availability of HIV services free from user fees; however, evidence shows that there remain gaps in who accesses these services. HIV outcome disparities occur based on sex, urban versus rural residence, age and wealth, among other factors.^1^ While some gaps in service access may relate to differences in residence area, income, age or access to education, many of the gaps may relate to structural elements or cultural norms which can impact access and health-seeking behaviours.

Achieving the Joint United Nations Programme on HIV and AIDS (UNAIDS) targets, which aim for 95% of people living with HIV to know their HIV status, 95% of those who know their status to be on treatment and 95% of those on treatment to attain viral load suppression (VLS), is crucial to ending the HIV epidemic.^2^ As we near these targets, it is critical to understand which, if any, subpopulations are being left behind and what is contributing to these gaps.^3^ Disparities between subpopulations represent missed opportunities to connect people with lifesaving treatment and limit new infections, hindering progress towards epidemic control.^4^

Gender disparities currently pose a barrier to achieving UNAIDS targets across the three indicators.^5^ One study found that females are nearly twice as likely as males to be aware of their status and have a suppressed viral load.^6^ Previous policy and programme recommendations have attempted to address barriers in HIV service delivery to address this gap.^4^,^7^ A descriptive paper using Population-based HIV Impact Assessments (PHIA) data on HIV services usage from 13 African countries found that HIV positive females were more likely than males: (1) to be aware of their positive status, (2) to have ever been on antiretroviral therapy (ART) treatment and (3) to achieve VLS. Even in countries with advanced epidemic control, such as Eswatini and Namibia, where VLS among females reaches as high as 77–82%, men’s rates are still approximately 7–8% lower.^1^ In an earlier study on HIV testing conducted in rural Zimbabwe, 78.7% of females had tested for HIV prior to the survey, compared with 66.1% of men, indicating that HIV testing coverage was significantly lower in males compared with females.^8^ This disparity in HIV outcomes is observed globally and is not confined to specific regions. A report on 15 President’s Emergency Plan for AIDS Relief (PEPFAR) countries found similar results, with males consistently performing more poorly than females across all three outcomes—awareness, treatment and VLS.^9^ This raises a critical question of determining which factors most strongly contribute to the gap. Identifying these key drivers is essential for designing and implementing services that effectively address gender disparities.

One study of HIV prevalence in young adults in Malawi, Tanzania and Zambia, using a Blinder-Oaxaca decomposition model for gender gaps, found that prevalence is higher for females than males, largely because they tend to live in urban areas and have older partners than do males.^10^ Both of these factors represent higher risk levels for females than they would be for comparable males. Young adults face unique challenges in the HIV care process. Research shows that females under age 25 are the least likely to be aware of their HIV status, while males aged 25–34, once aware, are the least likely to initiate ART.^11^ Additionally, males under 25 are the least likely to achieve VLS even after initiating ART, indicating that age, together with gender, influences outcomes of HIV service delivery. While this is an informative starting point, there is a need for greater understanding of what contributes to these disparities on both individual and societal levels. The present analysis expands on existing research by decomposing outcome disparities into explained and unexplained components. This decomposition provides insight into the extent to which disparities arise from individual-level characteristics versus structural or systemic factors.

This paper uses PHIA data for 13 African countries to examine gaps between adult males and females in HIV outcomes, specifically for awareness of HIV status, being on treatment and VLS. We use the Blinder-Oaxaca statistical method^12 13^ to decompose these gaps into three core elements: the portion of the gap due to observed differences in characteristics between the two groups, unobservable differences between the groups—most often attributed to structural barriers—and the residual or additional unexplained portion of the gender gap. The analysis includes results for the pooled sample of the 13 African countries included in this study, individual country results and analyses results over time for seven countries with two rounds of PHIA data.

## Methods

### Data

We used data from the 13 countries that were included in the first round of PHIA surveys conducted between 2015 and 2018, and seven countries that also have a second round of PHIA surveys. The PHIA project^14^ was initiated in 2014 and has since conducted surveys in 15 countries with high HIV epidemic rates to measure progress toward achieving the UNAIDS ‘95–95–95’ targets. The data collection was done through a cross-sectional survey that included interviews with household heads to gather household characteristics, and with household members, to assess individual characteristics, including those of adolescents, adults (age 15+) and in some surveys, children. The survey captures HIV outcomes such as awareness, treatment and VLS, as well as access to preventive measures and healthcare. Voluntary blood samples were collected for testing where applicable. The PHIA project is country-owned, with the Ministry of Health of the host country providing leadership with support from the International Center for AIDS Care and Treatment Programs (ICAP) at Columbia University.^14^

### Sample

The initial pooled dataset consisted of 13 African countries with PHIA surveys conducted between 2015 and 2018, including Cameroon (2017), Côte D'Ivoire (2017), Eswatini (2016), Ethiopia (2017), Kenya (2018), Lesotho (2016), Malawi (2015), Namibia (2017), Rwanda (2018), Tanzania (2016), Uganda (2016), Zambia (2016) and Zimbabwe (2015). The pooled dataset contained 302 355 observations. Second round surveys are included for Eswatini (2021), Lesotho (2020), Malawi (2020), Tanzania (2022), Uganda (2020), Zambia (2021) and Zimbabwe (2020). We included only respondents whose blood tests confirmed HIV-positive status (n=25 243). We then excluded all respondents with missing values in the relevant variables, resulting in a final analytical sample of 24 668 individuals, of whom 7807 were males, and 16 861 were females.

### Measures

We used three outcome measures as captured by PHIA surveys and blood tests. The first outcome measure, *awareness*, measured the share of people living with HIV who were aware of their status; the second outcome measure, *ART*, measured the share of people living with HIV who had ever been on antiretroviral treatment; the third outcome measure, *VLS*, measured the share of people living with HIV who had reached VLS. All outcome measures were operationalised as dichotomous variables. Covariates included dichotomous biological sex (male/female), age (continuous), a first wealth-quintile variable (dichotomous) to identify lowest-income status, a secondary or higher education variable (dichotomous) to identify high education level, employment in the past 12 months (dichotomous), ever being married (dichotomous) and urban/rural residence (dichotomous). The distribution of missing data for the variables of interest is as follows: HIV outcomes (awareness, ART and VLS)—417; wealth quintile—24; education—116; employment—15 and ever married—18.

### Statistical analysis

We used logit multivariate regression models to estimate the likelihood of the three outcome measures. Then, we used the Oaxaca-Blinder decomposition methodology to decompose the gender gap into three components: (1) the portion of the gap attributable to observed differences in characteristics between the two groups such as education or age (endowments portion), (2) the portion of the gap due to how the characteristics are related to the outcome (coefficients portion) and (3) the residual or unexplained portion of outcome differences which may reflect structural or behavioural factors such as stigma or cultural norms. This gap decomposition methodology is attributed to Blinder and Oaxaca and was selected because it enables quantification of how much of the gender gap is explained by measurable characteristics versus underlying structural factors.^12 13^ To account for heterogeneity and country-specific variation in the pooled model, we adjusted standard errors by clustering individuals within countries.

The equation to derive a measure of an expected outcome using the difference in outcome *Y* between males and females was:


$$\begin{array}{l} Mean\;(Y_{W})-Mean\;(Y_{M})=B_{W}\;Mean\;(X_{W})-B_{M}\;Mean\;(X_{M})\\ =B_{W}\;(Mean\;(X_{W})-Mean\;(X_{M})+Mean\;(X_{M})(B_{W}-B_{M}) \end{array}$$

Where *X_W_* and *X_M_* are the covariates, and *B_W_* and *B_M_* are regression coefficients measuring how the covariates influence the rate of *Y* for males and females, respectively. We presented pooled and weighted results for all 13 countries, along with individual country results, in figures with percentages in the results section. A significance level of 0.05 was used for all statistical tests. We conducted all analyses using STATA V.18.

### Patient and public involvement

Patients and the public were not involved in the design, conduct or dissemination of this research.

## Results

### Logistic multivariate models

The logistic regression examined factors associated with awareness of HIV status, being on treatment and VLS in a pooled model and by country. The model controls for urban versus rural residence, wealth quintile, education, age, ever being married and working outside the home in the past 12 months. The findings indicate that females are significantly more likely to be aware of their HIV status compared with males in the pooled analysis (adjusted OR (aOR)=1.61, p<0.01) and in each individual country, except in Côte d’Ivoire and Rwanda, where there was no significant difference. Several covariates led to higher outcomes in the pooled model, including rural residence (aOR=1.09, p<0.1), having secondary education (aOR=1.17, p<0.01) and being older than 25 (aOR=3.30, p<0.01), while working outside of the home led to lower results (aOR=0.77, p<0.01). Similarly, females show a higher likelihood of being on treatment in both the pooled results (aOR=1.48, p<0.01) and most individual countries, with the exception of Cameroon, Côte d’Ivoire, Eswatini, Rwanda and Zambia, where the differences were not significant. Several covariates contributed to higher outcomes in the pooled model, including rural residence (aOR=1.07, p<0.1), being older than 25 (aOR=2.85, p<0.01), while ever being married (aOR=0.91, p<0.1) and working outside of the home (aOR=0.75, p<0.01) led to lower outcomes. For VLS, females are also more likely to achieve viral suppression in the pooled analysis (aOR=1.46, p<0.01) in most countries, except in Cameroon and Ethiopia, where the results were not statistically significant. In the pooled results, rural residence (aOR=1.10, p<0.01) and being older than 25 (aOR=2.98, p<0.01) were associated with higher outcomes, while work outside the home (aOR=0.80, p<0.01) was associated with lower outcomes. See online supplemental tables A5–A7 for full results. Logistic regression results look at the gaps between males and females while controlling for other factors; the Blinder-Oaxaca portion of the model compares mean outcomes between the groups while breaking them down into what accounts for these gaps through a decomposition.

### Pooled results

**Figure 1:**
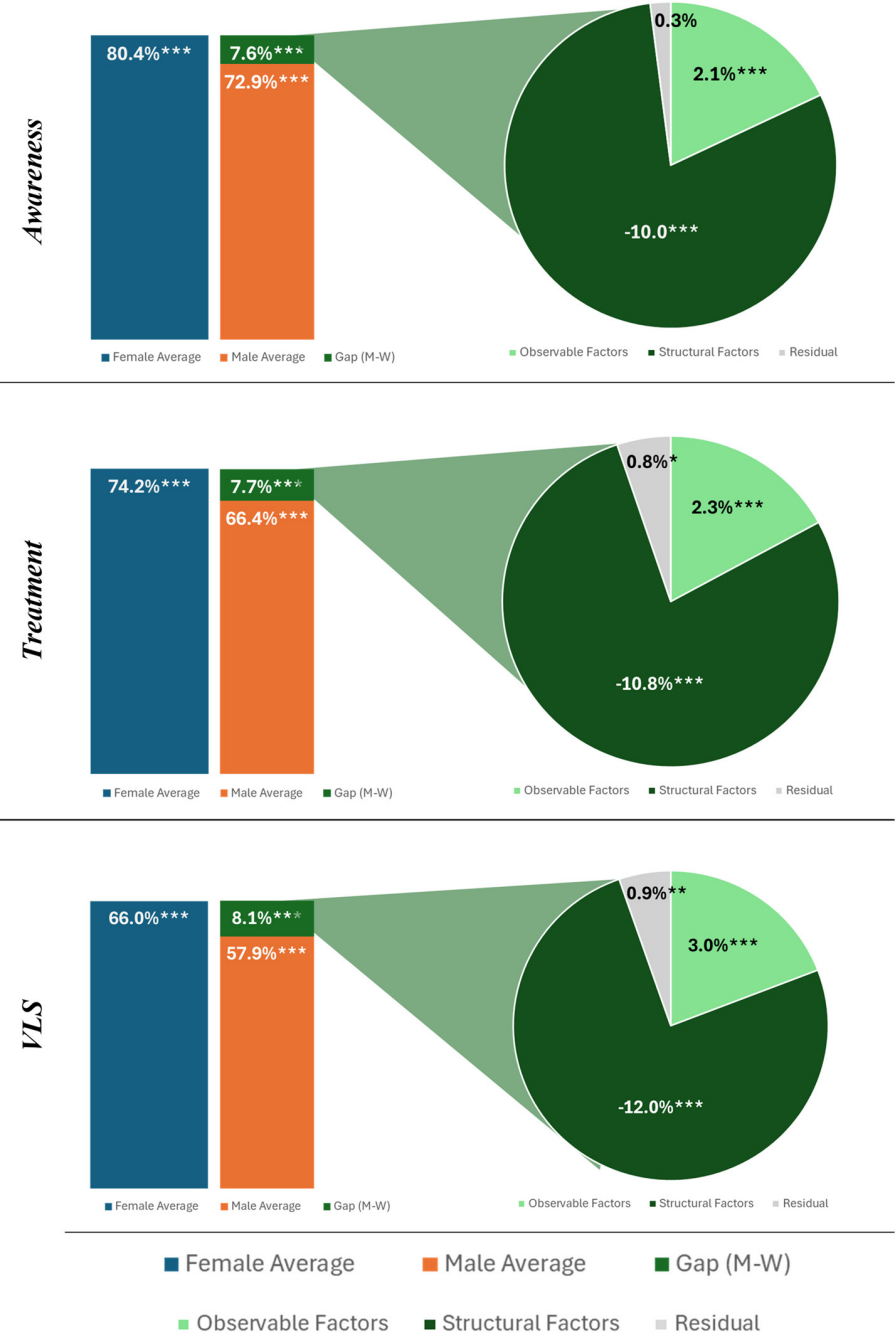
Pooled results decomposition of awareness, treatment and VLS outcomes. Source of data: 2015–2018 Population-based HIV Impact Assessment. *p<0.1, **p<0.05 and ***p<0.01. M-W, Men-Women; VLS, viral load suppression.

Pooled results show that, across all three indicators, males have lower average outcomes than females. When decomposing this gap, structural factors account for the largest contributor to lowered male outcomes. Males hold a higher level of protective characteristics (ie, age, wealth quintile and education) that would otherwise lead to higher HIV outcomes, as indicated through small positive values. However, large negative impacts from structural factors lead to worse performance among males across awareness of HIV positive status, being on treatment and viral suppression. Within awareness, males had 7.6 percentage points (pp) lower awareness of their HIV status when compared with females. Structural factors lead to males having 10.0 pp lower results than females, but the protective individual characteristics reduce this gap by 2.4 pp, resulting in the observed 7.6 pp disparity. In awareness, there was no significant residual. See online supplemental table A1 and figure 1 for a breakdown of pooled results.

The full pooled model results show that age, education and wealth endowments are positively related to increased levels of awareness of one’s HIV status, being on treatment and VLS. Conversely, working outside the home in the past 12 months and ever being married are negatively related to outcomes. Urban versus rural residence did not have a significant impact on outcomes. Taken together in the endowments portion of the model for awareness, these factors give males an estimated 2.3 pp advantage, due largely to advantages associated with older age, wealth and education, along with the slight disadvantage of working outside the home. The large negative values in the coefficients portion of the awareness model indicate that structural factors have a large negative impact on men. The models show that the factor with significantly more risk for females is being in the workplace.

Within treatment, there was a roughly 7.7 pp lower share of males on treatment when compared with females. Similar to awareness, this was primarily due to structural barriers (−10.8 pp), while individual characteristics (2.3 pp) and residual effects (0.8 pp) provided a small protective factor. Within VLS, males performed 8.1 pp worse than females, with structural barriers accounting for a majority of this gap (−12.0 pp). Individual characteristics (3.0 pp) and residual effects (0.9 pp) provided a slight protection, reducing the overall negative impact of structural factors. Data from the second round of PHIA surveys show that the gap between males and females decreased across all three indicators and that structural barriers or cultural norms continue to account for a majority of the remaining gap.

### Individual country results

**Figure 2:**
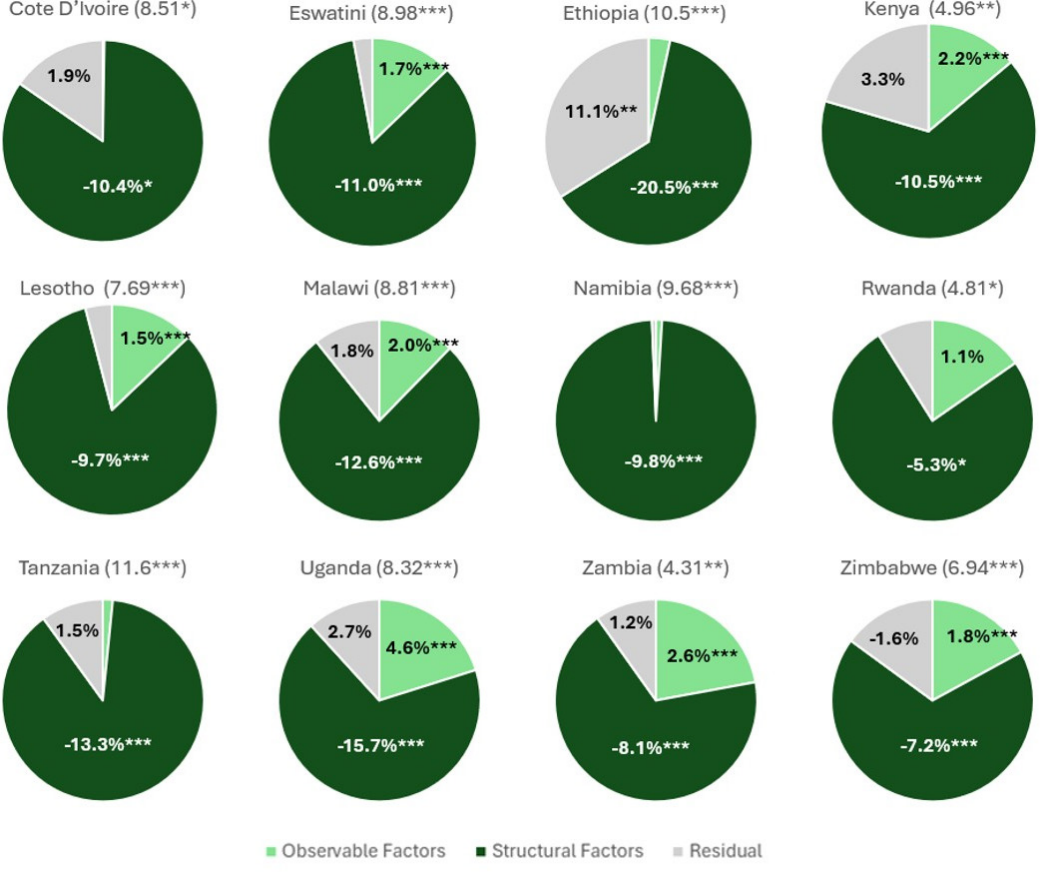
Awareness decomposition by country. Source of data: 2015–2018 Population-based HIV Impact Assessment. Numbers in parentheses indicate the percentage point outcome gaps between males and females; any results at or below 1% are shown without a numerical label; Cameroon was omitted since there was no significant outcome gap between males and females; *p<0.1, **p<0.05 and ***p<0.01.

**Figure 3:**
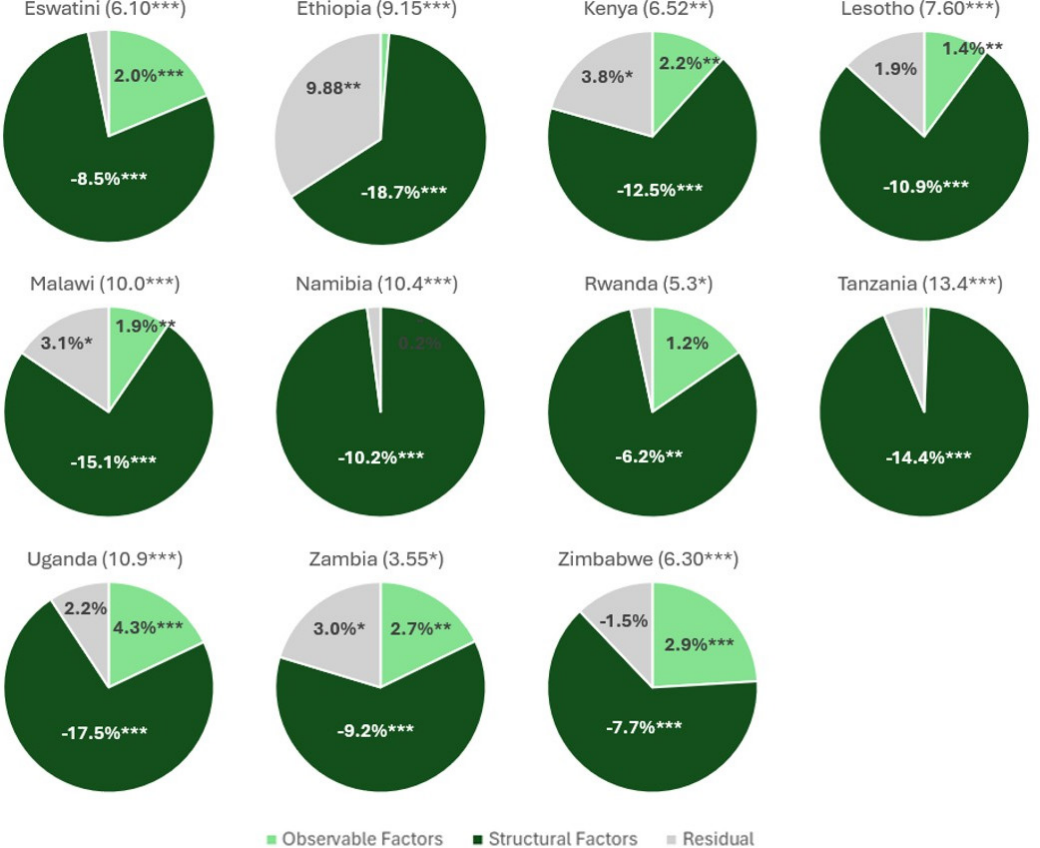
Treatment decomposition by country. Source of data: 2015–2018 Population-based HIV Impact Assessment. Numbers in parentheses indicate the percentage point outcome gaps between males and females; any results at or below 1% are shown without a numerical label; Cameroon and Côte d’Ivoire were omitted since there was no significant outcome gap between males and females; *p<0.1, **p<0.05 and ***p<0.01.

**Figure 4:**
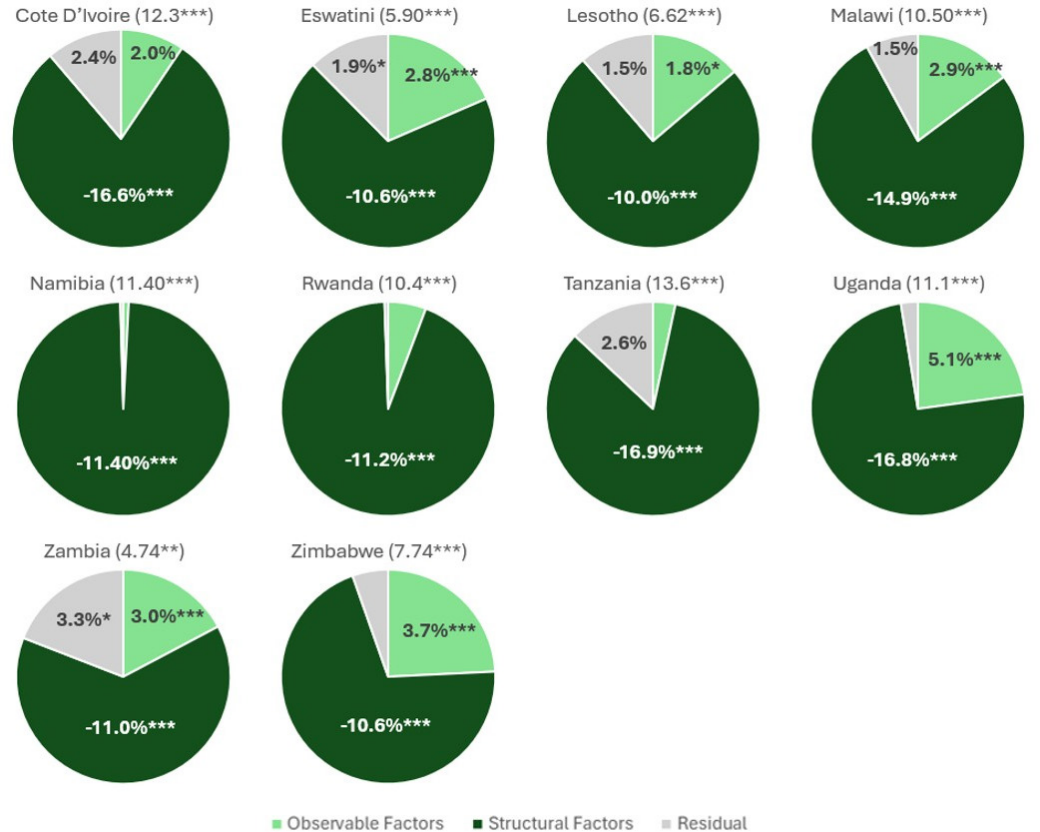
Viral load suppression decomposition by country. Source of data: 2015–2018 Population-based HIV Impact Assessment. Numbers in parentheses indicate the percentage point outcome gaps between males and females; any results at or below 1% are shown without a numerical label; Cameroon, Ethiopia and Kenya were omitted since there was no significant outcome gap between males and females; *p<0.1, **p<0.05 and ***p<0.01.

The analysis also provides individual country decomposition models for awareness, treatment and VLS (see online supplemental tables A2–A7 for detailed results, and figures 2–4). Tanzania has the largest gaps, with females performing better across awareness, share on treatment and VLS (11.6, 13.4, 13.6 pp, respectively). Cameroon is the only country without a significant gap between males and females across all three outcomes. Côte d’Ivoire does not have a significant difference between males and females in the share of treatment, while Ethiopia and Kenya have no significant difference between males and females in VLS. For all 13 countries with disparities, the basic pooled pattern of results holds: the gender gap favouring higher outcomes for females arises due to structural factors (coefficients). If other factors were the same, males would have a small advantage based on observable risk factors such as age, education and wealth, based on the endowments portion of the model. These findings reaffirm the pooled country results that structural factors (coefficients) are the main driving force behind these disparities.

### Awareness

All countries except Cameroon show greater rates of awareness among females than males, with the average gap being 7.6 pp (See online supplemental tables A2 and A5 and figure 2). Tanzania, Ethiopia and Namibia have the greatest gaps (11.6, 10.5 and 9.7 pp, respectively). The smallest gaps exist in Zambia and Rwanda (4.3 and 4.8 pp, respectively). Results in nine countries indicate that males held a greater number of observable characteristics that serve as protective factors, playing a role in lowering the gap. The endowments (observable) portion of the model was not significant in Ethiopia, Côte D’Ivoire, Namibia and Tanzania, indicating that observed characteristics were not a significant factor in disparities within these countries. Structural factors (coefficient results) across all countries with disparities provided an average 10.0 pp contribution to the gap in favour of females. The largest coefficient gaps favouring females were observed in Ethiopia, Uganda, Tanzania and Malawi (–20.5, –15.7, −13.3 and −12.6 pp, respectively). Rwanda and Zimbabwe have smaller coefficient margins favouring females at −5.3 and −7.2 pp, respectively. The residual output was significant in Ethiopia, at 11.1 pp.

### Treatment

The average gender gap in share on treatment is 7.7 pp. The largest gaps were found in Tanzania, Uganda, Namibia and Malawi (13.4, 10.9, 10.4 and 10.0 pp, respectively), while the smallest gaps were found in Zambia and Rwanda (3.6 and 5.3 pp, respectively). There was no significant outcome gap between males and females in Cameroon and Côte d’Ivoire. See online supplemental tables A3 and A6 and figure 3 for full results. Endowment results for the treatment gap are generally positive, indicating that these characteristics are more often observed in males, play a small role in increasing males’ average result, and thereby decrease the gender gap. Endowment results weren’t significant in Ethiopia, Namibia, Rwanda and Tanzania, indicating that differences in individual-level characteristics did not play a significant role in outcome differences between the two groups. All countries with treatment gaps show significant negative estimates of the portion of the treatment gap explained by coefficients (online supplemental tables A3 and A6), or unobservable factors such as discrimination or cultural norms. Like awareness, these results favour higher treatment rates for females. The coefficient portion of the model plays the strongest role in Ethiopia, Uganda and Malawi (18.7, 17.5 and 15.1 pp, respectively), showing that these factors play the strongest role in these countries. The coefficient, or structural, portion of the model plays the smallest role in Rwanda, Zimbabwe and Eswatini at 6.2, 7.7 and 8.5 pp, respectively. The residual impact was significant across pooled countries (0.81 pp), Ethiopia (9.88 pp), Kenya (3.81 pp), Malawi (3.12 pp) and Zambia (3.03 pp).

### Viral load suppression

The gender gap (males−females) in VLS is about 8.1 pp for all countries pooled together, with an average VLS rate of 58% for males and 66% for females. Tanzania, Côte d’Ivoire, Namibia and Uganda displayed the largest gaps, at 13.6, 12.3, 11.4 and 11.1 pp, respectively. There was no significant difference between VLS rates between males and females in Cameroon, Ethiopia and Kenya. The endowment portion of the model provided a roughly 3.07 pp advantage to males. Uganda and Zimbabwe showed the highest endowment results at 5.14 and 3.65 pp, respectively. Endowments did not play a significant role in VLS differences in Côte d’Ivoire, Namibia, Rwanda and Tanzania. The coefficient portion of the decomposition favours females by roughly −12.1 pp, with results significant across all countries. Tanzania, Uganda, Côte d’Ivoire and Malawi displayed the largest coefficient impact at 16.9, 16.8, 16.6 and 14.9 pp, respectively. Lesotho, Zimbabwe and Eswatini had the lowest results at 10.0, 10.6 and 10.6 pp, respectively. For all results, see online supplemental tables A4 and A7 and figure 4. The residual effect was significant in the pooled model (0.9 pp), Eswatini and Zambia (1.9 and 3.3 pp, respectively).

### Results over time

The seven countries with two rounds of PHIA survey data show that while outcomes for both males and females have improved, male outcomes have improved faster than females, which has led to a smaller gap across countries (see online supplemental tables A8–A10). These results show that in the 5–6 years between surveys, the gaps that had favoured women by between 7 pp and 8 pp reduced to only 3 – 5 pp.

Among the included countries, Tanzania made the largest improvements across each indicator, reducing the gender gap by 7.68, 8.87 and 7.93 pp for awareness, treatment and VLS, respectively. Tanzania had some of the lowest outcomes during PHIA 1, with only 53.8% of men aware of their status, 48.4% on treatment and only 40.8% virally suppressed, which increased to 82.9%, 80.9% and 75.4%, respectively. The gender gap decreased across countries, with the exception of Zambia, where treatment rates for women improved more quickly than men, leading to a slightly larger treatment gap between genders at 3.9 pp in PHIA 1 to 4.3 pp in PHIA 2.

**Figure 5:**
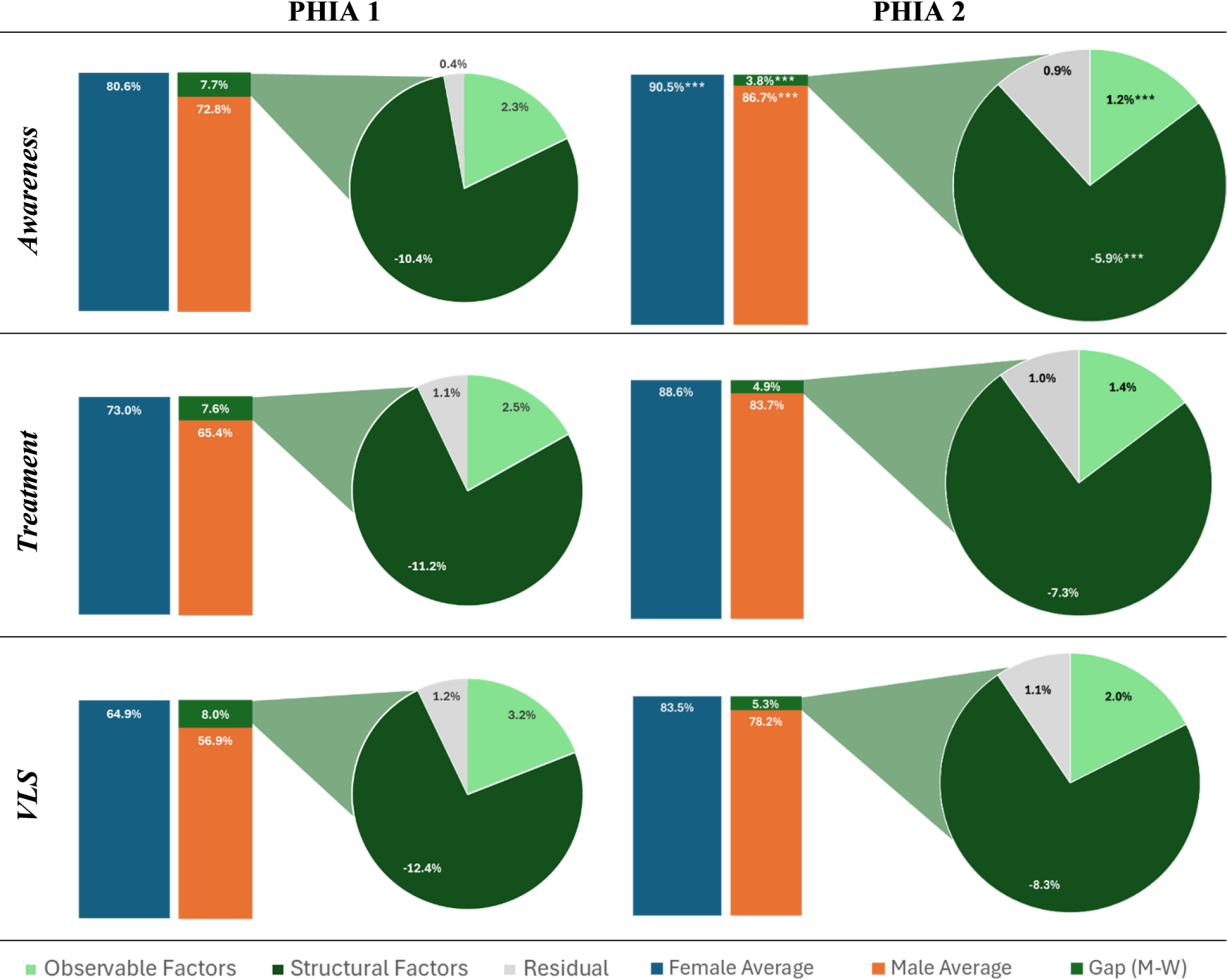
Pooled results decomposition over time. Source of data: 2015–2022 PHIA. *p<0.1, **p<0.05 and ***p<0.01. PHIA, Population-based HIV Impact Assessments; VLS, viral load suppression.

The endowments portion of the model decreased in all but one country, indicating that men and women share a higher level of individual-level characteristics (ie, wealth, age, education, etc) than during the first survey, and therefore these characteristics play less of a protective factor for men at this time. Similar to the round 1 PHIA surveys, structural barriers or cultural norms continue to be the largest contributor to the gender gap, as seen through the coefficients portion of the model. See figure 5.

## Discussion

### Factors impacting male treatment outcomes

The present analysis confirms that there is a significantly lower share of males living with HIV who are aware of their status, on treatment and have achieved VLS, when compared with females living with HIV. The exceptions to this are Cameroon across all three indicators, Côte d’Ivoire in treatment, and Ethiopia and Kenya in VLS, where there wasn’t a significant gap. Beyond this, the analysis confirms that in countries with gaps, males tend to carry more protective individual-level factors such as higher wealth, age and levels of education than females; however, structural barriers are likely to lead to worse outcomes for males. These findings show that, if individual characteristics between the groups were the same, the observed gaps would be larger across countries with disparities. This suggests that structural challenges, whether they are discrimination, cultural norms or other unobservable factors, may drive lower outcomes among men. These disparities represent a significant barrier to achieving UNAIDS 95–95–95 targets and ending the HIV epidemic. Addressing structural barriers can be a challenge, as it often involves behaviour change, cultural norms, addressing deeply rooted ideas about personal health decisions, or other factors that are more difficult to measure. Recent work has shown that while the HIV outcome gap between males and females has reduced over time, there remains a need to understand the factors that contribute to this disparity, along with effective solutions.^5^

### Understanding structural factors

Previous research has shown that certain norms and beliefs can impact health-seeking behaviour, including the idea that males are expected to display more ‘masculine’ traits or mindsets. Researchers in Zimbabwe observed that this mindset was characterised by the ability to be in control, be financially reliable, resilient, healthy and, at times, engage in risky sexual behaviour.^15^ These traits can be at odds with traits needed to prevent and treat HIV, such as consistent condom use, regular testing for HIV or sexually transmitted infections, and adherence to treatment. These factors may also impact willingness to access services or follow medical advice from nurses, a female-dominated profession.^15^ This is backed by a UNAIDS study in Mpumalanga, South Africa, where 26% of males perceived seeking healthcare as a sign of weakness and tended to overestimate the extent to which their peers shared this view.^16^ Other studies have shown in communities where males face economic hardships, males are more likely to engage in transactional sex as it is a perceived venue to assert their masculinity. In contrast, for females, transactional sex is often driven by financial need, emotional connection, social status or the desire to maintain a modern lifestyle.^17^

Beyond these behaviours, there may be gaps in knowledge. Almost half (47%) of males aged 18–30 in South Africa who partnered with females reported perceiving no chance of getting HIV in their lifetime.^17^ This is shown through risky behaviours, as males who reported inconsistent condom use and transactional sex were more likely to also have concurrent sexual partnerships, perpetrate intimate partner violence and believe they had no chance of acquiring HIV.^17^ Beyond this, fears of stigma, clinic wait times, confidentiality, limited clinic hours and fear of testing positive serve as barriers to accessing testing and care.^18^

UNAIDS’ comprehensive strategy notes that due to gender norms that emphasise resilience and independence, males exhibit poorer health-seeking behaviour, leading to lower HIV awareness and treatment rates, with the result that males receiving ART face a 70% higher mortality risk from HIV compared with females.^16^

Poorer outcomes among adult males have rippling effects. When someone living with HIV knows their status, is on treatment and achieves VLS, they can no longer transmit the virus to a loved one through sexual or blood contact.^19^ Low VLS among adult males represents a missed opportunity for leveraging treatment as prevention. Adolescent girls and young women in sub-Saharan Africa are more likely to have a sexual partner who is five or more years older than their male peers.^10^ As such, low VLS coverage among males is a key contributor to the disproportionate number of new infections among adolescent girls and young women, who are three times more likely to acquire HIV than young men.^5^ Increasing testing, treatment and retention in care for males is key to supporting individual health, preventing new infections and ending the HIV epidemic.

### Programmatic strategies to reduce disparities

While this evidence shows how beliefs and structural factors lower health-seeking behaviour among men, there is also evidence that tailored programming can help to breach this gap.^15 20^ A study in Zimbabwe showed that, after receiving education on the lifesaving benefits of ART and its impact on their well-being, some males adjusted their perceptions of masculinity and became more adherent to ART.^15^ Early communications on HIV often provided stigmatising messages, highlighting illness or stigmatising the behaviours of people living with HIV.^21^ Stigma is directly linked to lower rates of testing, accessing treatment and remaining on treatment once started.^21^ This research shows that shifting towards messaging that highlights the lifesaving benefits of treatment and that one can no longer transmit the virus to loved ones when you reach viral suppression (undetectable=untransmittable U=U)^19^ may play a role in both reversing this stigma and creating a positive perception of HIV testing and treatment.

Historically, much of the global HIV response in Africa focused on females due to higher perceived risk, and as part of very successful prevention of mother-to-child transmission programmes.^22^ However, an increasing number of interventions are being tailored to target men.

### Improving male outcomes over time

Countries with two rounds of PHIA data showed significant improvements among males and a corresponding reduction in the gender gap, with the largest improvements in Malawi, Tanzania and Uganda. In each of these, structural or cultural barriers (from the coefficients portion of the model) were the largest contributor to this gap across both surveys; however, they played a much smaller role during PHIA 2. While there is a need for formal evaluations of these interventions, each of these countries has attempted to tailor services to overcome barriers to care for men.

To increase awareness, each of these countries has scaled up HIV self-testing in various venues such as pharmacies, workplaces, faith communities or public spaces (such as vending machines, in Uganda).^23 24^ The social network strategy in Malawi and Tanzania, among other countries, engages ‘recruiters’ to encourage people in their social networks, whether friends, sexual partners, family or part of the same faith community, to get tested.^25^,^28^ These interventions are paired with recency testing, which allows for targeted testing and prevention services in areas with high rates of new infections.

The PEPFAR Faith and Community Initiative attempted to work with community leaders, faith-based organisations and faith communities to reach men, replacing previous stigmatising messages with messaging around the lifesaving role of testing and treatment.^29^ These countries, among others, have also implemented strategies to ensure that treatment is accessible for men. Male-friendly clinics or corners have been found to support higher levels of adherence and have been implemented across each of these countries.^23^,^2630^ These are often characterised by expanded hours, peer support from male expert clients,^33^ being a one-stop shop to ensure minimal time is spent in the appointment, and often engaging male healthcare workers. For those who drop out of treatment, Tanzania has employed peer counsellors who follow-up with clients for appointment reminders and peer counselling, community ART groups for peer support to encourage them to engage in care.^26^

While the above interventions provide a starting point, there remains a critical need for formal evaluation of these programmes to assess their relative effectiveness and overall impact. Such evaluations are essential for guiding future programme design and budget allocation, with the goal of further narrowing the outcome disparities between males and females. However, future investments in these interventions are uncertain due to political pressure to reduce PEPFAR funding, along with the recent dismantling of the US Agency for International Development, one of the programme’s primary implementing agencies.^34 35^ Conducting rigorous evaluations of male-friendly service models will be vital to inform and prioritise future investments, ensuring continued progress in closing gender gaps despite constrained budgetary environments.

### Limitations

Ethiopia does not have data on rural versus urban residence, so its results were not broken down in this regard. The residual value in Ethiopia was larger than in any other country. While this may be partially attributable to the lack of data on urban versus rural residence, further research is needed to understand which factors weren’t captured in the current model to better understand if additional individual-level characteristics impact service outcomes in the country.

Additionally, the above analysis highlights key disparities between male and female HIV outcomes, yet further analysis is needed to understand which programme and policy interventions are most successful in addressing these gaps. Future studies may evaluate these interventions to understand which have been most effective in increasing HIV service uptake among men.

## Conclusion

This study confirms that across 12 out of 13 countries studied, females perform better than males across outcomes of awareness of one’s HIV status, being on treatment and VLS. The study confirms that a majority of this gap is attributable to structural barriers to care. While males as a whole hold individual-level characteristics that would otherwise increase their expected outcomes, the larger role of these structural barriers reinforces this gender gap. Policymakers and programme managers should consider how cultural norms, ideas towards health-seeking behaviour and other structural factors may impact males' access to services. Continued investment in and evaluation of male-friendly services is vital to decrease this gap and understand effective strategies to link males to HIV care. Future studies may explore existing approaches to increase evidence-based strategies to improve HIV outcomes.

## Supplementary material

10.1136/bmjgh-2025-020900 online supplemental file 1

## Data Availability

Data are available upon reasonable request.
