# Supplementary material for: Decomposing gender gaps in HIV service outcomes
Source: BMJ Glob Health. 2026 Jan 16;11(1):e020900. doi: 10.1136/bmjgh-2025-020900 (PMC12815156; doi:10.1136/bmjgh-2025-020900)
Supplement: online supplemental file 1 [file bmjgh-11-1-s001.docx]

**Annex**

**Table A1. Pooled Blinder-Oaxaca Analysis of Gender Gap in Awareness, Treatment and VLS**

|  | **Awareness** | | | **Treatment** | | | **VLS** | | |
| --- | --- | --- | --- | --- | --- | --- | --- | --- | --- |
| **Overall** | | | | | | | | | |
|  |  | | |  | | |  | | |
| Men | .729*** | | | .664*** | | | .579*** | | |
|  | (.024) | | | (.024) | | | (.026) | | |
| Women | .804*** | | | .742*** | | | .660*** | | |
|  | (.024) | | | (.024) | | | (.024) | | |
| Gap | -.076*** | | | -.077*** | | | -.081*** | | |
|  | (.006) | | | (.008) | | | (.009) | | |
| Endowments | .021*** | | | .023*** | | | .030*** | | |
|  | (.004) | | | (.004) | | | (.004) | | |
| Coefficients | -.100*** | | | -.108*** | | | -.120*** | | |
|  | (.007) | | | (.009) | | | (.008) | | |
| Interaction | .003 | | | .008* | | | .009** | | |
|  | (.004) | | | (.005) | | | (.005) | | |
| **Covariates** | | | | | | | | | |
|  | **Endowments** | **Coefficient** | **Interaction** | **Endowments** | **Coefficient** | **Interaction** | **Endowments** | **Coefficient** | **Interaction** |
| Age | .023*** | .052** | -.003 | .028*** | .110*** | .032 | .033*** | .118*** | .019*** |
|  | (.001) | (.024) | (.008) | (.002) | (.027) | (.026) | (.002) | (.026) | (.004) |
| Wealth Quintile | .001*** | .054*** | .001 | .001*** | .078*** | -.007 | .001*** | .057*** | -.003** |
|  | (.000) | (.016) | (.002) | (.000) | (.018) | (.007) | (.000) | (.018) | (.001) |
| Education | .002*** | -.016 | .000 | .001*** | -.011 | -.001 | .001*** | -.004 | -.000 |
|  | (.000) | (.011) | (.001) | (.000) | (.011) | (.002) | (.000) | (.012) | (.001) |
| Work in 12 months | -.003*** | -.015*** | .004 | -.006*** | -.011** | -.015 | -.005*** | -.007 | -.005 |
|  | (.001) | (.005) | (.008) | (.001) | (.005) | (.020) | .001) | (.005) | (.005) |
| Evermarried | -.000 | -.009 | .000 | -.000 | -.042*** | -.004 | -.000 | -.033** | -.002* |
|  | (.000) | (.014) | (.001) | (.000) | (.015) | (.004) | (.000) | (.016) | (.001) |
| Urban rural | .000 | .001 | -.000 | -.000 | .006 | .002 | .001 | -.004 | -.001 |
|  | (.000) | (.008) | (.001) | (.000) | (.010) | (.003) | (.001) | (.009) | (.001) |
| Constant |  | -.168*** |  |  | -.239*** |  |  | -.248*** |  |
|  |  | (.034) |  |  | (.037) |  |  | (.038) |  |
|  |  |  |  |  |  |  |  |  |  |
| Observations | 24,668 | 24,668 | 24,668 | 24,668 | 24,668 | 24,668 | 24,668 | 24,668 | 24,668 |

Notes: Pooled Across 13 Countries: Cameroon, Cote D'Ivoire, Eswatini, Ethiopia, Kenya, Lesotho, Malawi, Namibia, Rwanda, Tanzania, Uganda, Zambia, Zimbabwe. VLS=viral load suppression. Standard errors in parentheses. * p<0.1, ** p<0.05, *** p<0.01.

**Table A2. Blinder-Oaxaca Decomposition by Country for Awareness Outcomes**

| Country | Male | Female | Difference | Endowments | Coefficients | Interaction | n |
| --- | --- | --- | --- | --- | --- | --- | --- |
| Pooled | .729*** (.024) | .804***  (.024) | -.076*** (.006) | .021***  (.004) | -.100*** (.007) | .003  (.004) | 24,668 |
| Cameroon | .512***  (.029) | .555***  (.019) | -.043  (.035) | .068*** (.015) | -.103*** (.040) | -.007  (.026) | 972 |
| Cote D’Ivoire | .446***  (.042) | .531***  (.029) | -.085*  (.051) | .000  (.020) | -.104*  (.055) | .019  (.030) | 444 |
| Eswatini | .823***  (.012) | .913***  (.006) | -.090*** (.014) | .017*** (.003) | -.110*** (.016) | .004  (.007) | 2,981 |
| Ethiopia | .727***  (.037) | .831***  (.018) | -.105*** (.041) | -.011  (.017) | -.205*** (.061) | .111**  (.047) | 601 |
| Kenya | .769***  (.021) | .819***  (.012) | -.050**  (.024) | .022***  (.008) | -.105*** (.032) | .033  (.020) | 1,449 |
| Lesotho | .773***  (.013) | .850***  (.008) | -.077*** (.015) | .015*** (.005) | -.097*** (.018) | .005  (.011) | 3,188 |
| Malawi | .716***  (.017) | .804***  (.010) | -.088*** (.020) | .020*** (.008) | -.126*** (.025) | .018  (.016) | 2,215 |
| Namibia | .811***  (.014) | .908***  (.007) | -.097*** (.016) | .001  (.004) | -.098*** (.017) | .002  (.008) | 2,427 |
| Rwanda | .813***  (.022) | .861***  (.014) | -.048*  (.026) | .011  (.008) | -.053*  (.028) | -.006  (.014) | 931 |
| Tanzania | .538***  (.021) | .654***  (.014) | -.116*** (.025) | .002  (.010) | -.133*** (.029) | .015  (.018) | 1,780 |
| Uganda | .681***  (.020) | .764***  (.012) | -.083*** (.023) | .046*** (.009) | -.157*** (.028) | .027  (.018) | 1,760 |
| Zambia | .698***  (.017) | .741***  (.011) | -.043**  (.020) | .026***  (.009) | -.081*** (.024) | .012  (.016) | 2,418 |
| Zimbabwe | .751***  (.012) | .820***  (.008) | -.069*** (.015) | .018***  (.005) | -.072*** (.017) | -.016  (.010) | 3,502 |

Notes: Standard errors in parentheses. * p<0.1, ** p<0.05, *** p<0.01.

**Table A3. Blinder-Oaxaca Decomposition by Country for Treatment Outcomes**

| Country | Male | Female | Difference | Endowments | Coefficients | Interaction | n |
| --- | --- | --- | --- | --- | --- | --- | --- |
| Pooled | .664***  (.024) | .742***  (.024) | -.077***  (.008) | .023***  (.004) | -.108***  (.009) | .008*  (.005) | 24,668 |
| Cameroon | .481***  (.029) | .515***  (.019) | -.034  (.035) | .065***  (.016) | -.092**  (.040) | -.008  (.026) | 972 |
| Cote D’Ivoire | .410***  (.042) | .492***  (.029) | -.082  (.051) | .006  (.020) | -.120**  (.054) | .032  (.031) | 444 |
| Eswatini | .749***  (.014) | .810***  (.009) | -.061***  (.017) | .020***  (.005) | -.085***  (.018) | .003  (.009) | 2,981 |
| Ethiopia | .713***  (.037) | .805***  (.019) | -.092**  (.042) | -.004  (.017) | -.187***  (.062) | .099** (.047) | 601 |
| Kenya | .724***  (.022) | .790***  (.013) | -.065**  (.026) | .022**  (.009) | -.125***  (.033) | .038*  (.021) | 1,449 |
| Lesotho | .710***  (.014) | .786***  (.009) | -.076***  (.017) | .014**  (.006) | -.109***  (.019) | .019  (.012) | 3,188 |
| Malawi | .638***  (.018) | .738***  (.011) | -.100***  (.021) | .019**  (.009) | -.151***  (.026) | .031*  (.017) | 2,215 |
| Namibia | .775***  (.015) | .879***  (.008) | -.104***  (.017) | .000  (.005) | -.102***  (.018) | -.002  (.009) | 2,427 |
| Rwanda | .786***  (.023) | .839***  (.015) | -.053*  (.027) | .012  (.009) | -.062**  (.030) | -.003  (.015) | 931 |
| Tanzania | .484***  (.021) | .617***  (.014) | -.134***  (.025) | .001  (.011) | -.144***  (.029) | .010  (.018) | 1,780 |
| Uganda | .595***  (.021) | .704***  (.013) | -.109***  (.024) | .043***  (.010) | -.175***  (.028) | .022  (.018) | 1,760 |
| Zambia | .612***  (.018) | .648***  (.012) | -.036*  (.021) | .027**  (.010) | -.092***  (.025) | .030*  (.017) | 2,418 |
| Zimbabwe | .671***  (.014) | .734***  (.009) | -.063***  (.016) | .029***  (.006) | -.077***  (.019) | -.015  (.011) | 3,502 |

Notes: Standard errors in parentheses. * p<0.1, ** p<0.05, *** p<0.01.

**Table A4. Blinder-Oaxaca Decomposition by Country for VLS Outcomes**

| Country | Male | Female | Difference | Endowments | Coefficients | Interaction | n |
| --- | --- | --- | --- | --- | --- | --- | --- |
| Pooled | .579***  (.026) | .660***  (.024) | -.081*** (.009) | .030***  (.004) | -.120*** (.008) | .009**  (.005) | 24,668 |
| Cameroon | .377***  (.028) | .410***  (.019) | -.033  (.034) | .058***  (.016) | -.096*** (.036) | .004  (.024) | 972 |
| Cote D’Ivoire | .273***  (.038) | .397***  (.028) | -.123*** (.047) | .020  (.021) | -.166*** (.046) | .024  (.028) | 444 |
| Eswatini | .683***  (.015) | .742***  (.010) | -.059*** (.018) | .028***  (.006) | -.106*** (.020) | .019*  (.010) | 2,981 |
| Ethiopia | .647***  (.039) | .692***  (.022) | -.045  (.045) | .008  (.020) | -.117*  (.062) | .064  (.046) | 601 |
| Kenya | .669***  (.024) | .713***  (.014) | -.044  (.027) | .033***  (.011) | -.119*** (.034) | .042**  (.021) | 1,449 |
| Lesotho | .623***  (.015) | .690***  (.010) | -.066*** (.018) | .018**  (.007) | .100***  (.020) | .015  (.012) | 3,188 |
| Malawi | .573***  (.019) | .678***  (.012) | -.105*** (.022) | .029***  (.010) | -.149*** (.026) | .015  (.017) | 2,215 |
| Namibia | .685***  (.017) | .799***  (.010) | -.114*** (.020) | .001  (.006) | -.114*** (.021) | -.001  (.010) | 2,427 |
| Rwanda | .676***  (.027) | .780***  (.017) | -.104*** (.032) | .007  (.010) | -.112*** (.034) | .001  (.017) | 931 |
| Tanzania | .408***  (.021) | .543***  (.014) | -.136*** (.025) | .007  (.011) | -.169*** (.028) | .026  (.018) | 1,780 |
| Uganda | .481***  (.021) | .592***  (.014) | -.111***  (.025) | .051***  (.011) | -.168*** (.028) | .006  (.017) | 1,760 |
| Zambia | .537***  (.018) | .584***  (.012) | -.047**  (.022) | .030***  (.011) | -.110*** (.025) | .033*  (.017) | 2,418 |
| Zimbabwe | .567***  (.014) | .645***  (.010) | -.077*** (.017) | .037***  (.007) | -.106*** (.020) | -.008  (.011) | 3,502 |

Notes: VLS=viral load suppression. Standard errors in parentheses. * p<0.1, ** p<0.05, *** p<0.01.

**Table A5. Logistic Model Results with Adjusted Odds Ratio by Country for Awareness Outcomes**

| Country | Gender (Female=1) | Urban/Rural (Rural=1) | Wealth Quintile (WQ1=1) | Education (>=Secondary=1) | Age (Age>=25=1) | Ever Married (Married=1) | Work Outside Home Last 12 Months (yes=1) |
| --- | --- | --- | --- | --- | --- | --- | --- |
| Pooled | 1.608*** | 1.089* | 1.035 | 1.169*** | 3.297*** | 0.931 | 0.774*** |
|  | (1.506-1.716) | (1.018-1.164) | (0.954-1.124) | (1.096-1.248) | (3.001-3.622) | (0.855-1.013) | (0.726 -0.825) |
| Cameroon | 1.404* | 1.144 | 0.485*** | 1.08 | 4.834*** | 1.12 | 1.081 |
|  | (1.042-1.890) | (0.855-1.530) | (0.333- 0.706) | (0.820-1.424) | (3.038-7.692) | (0.755-1.662) | (0.818-1.430) |
| Cote D’Ivoire | 1.419 | 0.863 | 1.36 | 1.19 | 3.354** | 0.885 | 1.097 |
|  | (0.933-2.157) | (0.564-1.321) | (0.782-2.367) | (0.693-2.043) | (1.508-7.459) | (0.527-1.486) | (0.746-1.613) |
| Eswatini | 2.708*** | 1.316* | 1.295 | 0.752* | 3.163*** | 1.811*** | 0.874 |
|  | (2.127-3.449) | (1.009-1.716) | (0.953-1.761) | (0.586-0.966) | (2.299-4.350) | (1.403-2.337) | (0.683-1.119) |
| Ethiopia | 1.776* |  | 0.999 | 1.055 | 3.404** | 0.706 | 0.77 |
|  | (1.126-2.802) | (NA) | (0.571-1.747) | (0.671-1.661) | (1.622-7.141) | (0.321-1.556) | (0.502-1.180) |
| Kenya | 1.438* | 1.144 | 0.917 | 1.169 | 2.211*** | 1.155 | 0.91 |
|  | (1.075-1.924) | (0.872-1.501) | (0.672-1.251) | (0.610-2.239) | (1.391-3.515) | (0.764-1.747) | (0.696-1.190) |
| Lesotho | 1.764*** | 0.996 | 0.807 | 0.95 | 2.621*** | 1.248 | 0.703*** |
|  | (1.449-2.147) | (0.803-1.236) | (0.622-1.049) | (0.774-1.167) | (1.973-3.483) | (0.974-1.599) | (0.577-0.857) |
| Malawi | 1.66*** | 1.199 | 0.803 | 0.951 | 3.05*** | 1.444 | 0.766* |
|  | (1.333-2.068) | (0.956-1.502) | (0.572-1.127) | (0.753-1.201) | (2.214-4.200) | (0.988-2.112) | (0.615-0.954) |
| Namibia | 2.285*** | 1.283 | 1.064 | 0.797 | 3.16*** | 1.132 | 0.597*** |
|  | (1.762-2.964) | (0.966-1.705) | (0.775-1.460) | (0.614-1.035) | (2.224-4.489) | (0.869-1.475) | (0.456-0.782) |
| Rwanda | 1.379 | 0.645* | 1.045 | 1.709* | 2.504*** | 1.813* | 0.616* |
|  | (0.940-2.022) | (0.426-0.977) | (0.627-1.741) | (1.012-2.886) | (1.452-4.320) | (1.127-2.916) | (0.424-0.896) |
| Tanzania | 1.573*** | 0.828 | 0.647** | 1.09 | 2.176*** | 1.262 | 0.76** |
|  | (1.269-1.949) | (0.667-1.028) | (0.497-0.844) | (0.776-1.532) | (1.538-3.079) | (0.882-1.807) | (0.622-0.928) |
| Uganda | 1.854*** | 0.927 | 0.997 | 0.966 | 3.966*** | 1.243 | 1.177 |
|  | (1.460-2.354) | (0.726-1.184) | (0.746-1.334) | (0.752-1.241) | (2.894-5.434) | (0.828-1.866) | (0.937-1.479) |
| Zambia | 1.33** | 0.704*** | 0.827 | 1.149 | 3.205*** | 1.749*** | 0.839 |
|  | (1.081-1.636) | (0.572-0.865) | (0.602-1.135) | (0.941-1.401) | (2.397-4.285) | (1.324-2.311) | (0.686-1.025) |
| Zimbabwe | 1.516*** | 1.077 | 1.066 | 0.852 | 3.2*** | 0.878 | 0.738*** |
|  | (1.272-1.807) | (0.885-1.311) | (0.857-1.324) | (0.712-1.020) | (2.409-4.249) | (0.643-1.200) | (0.620-0.879) |

Note: Confidence interval in parentheses. * p<0.1, ** p<0.05, *** p<0.01.

**Table A6. Logistic Model Results with Adjusted Odds Ratio by Country for Treatment Outcomes**

| Country | Gender (Female=1) | Urban/Rural (Rural=1) | Wealth Quintile (WQ1=1) | Education (>=Secondary=1) | Age (Age>=25=1) | Ever Married (Married=1) | Work Outside Home Last 12 Months (yes=1) |
| --- | --- | --- | --- | --- | --- | --- | --- |
| Pooled | 1.48*** | 1.07* | 1.025 | 1.022 | 2.853*** | 0.911* | 0.75*** |
|  | (1.393-1.572) | (1.006-1.137) | (0.950-1.105) | (0.964-1.085) | (2.606-3.124) | (0.842-0.985) | (0.707-0.795) |
| Cameroon | 1.333 | 1.096 | 0.514*** | 1.066 | 4.865*** | 1.258 | 1.042 |
|  | (0.992-1.793) | (0.822-1.462) | (0.353-0.750) | (0.810-1.402) | (2.996-7.900) | (0.847-1.868) | (0.789-1.375) |
| Cote D’Ivoire | 1.437 | 0.946 | 1.214 | 1.33 | 3.202** | 0.912 | 1.09 |
|  | (0.943-2.189) | (0.617-1.449) | (0.701-2.101) | (0.776-2.279) | (1.415-7.244) | (0.545-1.528) | (0.742-1.603) |
| Eswatini | 1.537 | 1.19 | 1.196*** | 0.722 | 2.25*** | 1.521 | 0.761 |
|  | (1.269-1.860) | (0.963-1.470) | (0.948-1.509) | (0.596-0.876) | (1.716-2.950) | (1.248-1.853) | (0.629-0.920) |
| Ethiopia | 1.578* |  | 1.015 | 1.015 | 2.767** | 0.746 | 0.785 |
|  | (1.015-2.453) | (NA) | (0.594-1.733) | (0.659-1.563) | (1.342-5.707) | (0.351-1.588) | (0.523-1.179) |
| Kenya | 1.511** | 1.156 | 0.824 | 1.296 | 2.294*** | 1.166 | 0.871 |
|  | (1.148-1.988) | (0.893-1.494) | (0.617-1.102) | (0.690-2.434) | (1.467-3.588) | (0.787-1.728) | (0.676-1.123) |
| Lesotho | 1.556*** | 1.067 | 0.873 | 0.981 | 2.17*** | 1.23 | 0.708*** |
|  | (1.305-1.856) | (0.882-1.292) | (0.690-1.105) | (0.818-1.177) | (1.665-2.829) | (0.983-1.538) | (0.594-0.844) |
| Malawi | 1.602*** | 1.296* | 0.789 | 0.936 | 2.696*** | 1.469* | 0.75** |
|  | (1.308-1.961) | (1.054-1.592) | (0.578-1.078) | (0.756-1.159) | (1.977-3.676) | (1.022-2.110) | (0.613-0.917) |
| Namibia | 2.062*** | 1.25 | 1.105 | 0.742* | 2.629*** | 0.997 | 0.586*** |
|  | (1.626-2.615) | (0.966-1.619) | (0.829-1.473) | (0.585-0.940) | (1.879-3.679) | (0.784-1.266) | (0.459-0.747) |
| Rwanda | 1.377 | 0.672* | 1.124 | 1.622 | 2.344** | 1.985** | 0.639* |
|  | (0.957-1.981) | (0.455-0.992) | (0.686-1.841) | (0.997-2.637) | (1.390-3.953) | (1.269-3.107) | (0.449-0.911) |
| Tanzania | 1.627*** | 0.815 | 0.65** | 1.06 | 2.222*** | 1.183 | 0.672*** |
|  | (1.316-2.013) | (0.658-1.008) | (0.499-0.846) | (0.758-1.482) | (1.567-3.152) | (0.827-1.691) | (0.552-0.819) |
| Uganda | 1.837*** | 0.957 | 0.837 | 0.789* | 3.281*** | 1.128 | 1.084 |
|  | (1.471-2.293) | (0.763-1.201) | (0.640-1.094) | (0.627-0.995) | (2.406-4.473) | (0.761-1.672) | (0.877-1.342) |
| Zambia | 1.201 | 0.747** | 0.748 | 1.126 | 2.975*** | 1.595*** | 0.787* |
|  | (0.991-1.455) | (0.617-0.904) | (0.555-1.010) | (0.938-1.352) | (2.231-3.967) | (1.218-2.088) | (0.654-0.947) |
| Zimbabwe | 1.332*** | 1.107 | 1.052 | 0.829* | 2.724*** | 0.786 | 0.729*** |
|  | (1.138-1.559) | (0.930-1.319) | (0.868-1.274) | (0.708-0.972) | (2.081-3.564) | (0.589-1.048) | (0.624-0.851) |

Note: Confidence interval in parentheses. * p<0.1, ** p<0.05, *** p<0.01.

**Table A7. Logistic Model Results with Adjusted Odds Ratio by Country for VLS Outcomes**

| Country | Gender (Female=1) | Urban/Rural (Rural=1) | Wealth Quintile (WQ1=1) | Education (>=Secondary=1) | Age (Age>=25=1) | Ever Married (Married=1) | Work Outside Home Last 12 Months (yes=1) |
| --- | --- | --- | --- | --- | --- | --- | --- |
| Pooled | 1.459*** | 1.104*** | 0.971 | 1.041 | 2.984*** | 0.965 | 0.795*** |
|  | (1.378-1.544) | (1.043-1.170) | (0.905-1.041) | (0.985-1.100) | (2.728-3.263) | (0.897-1.038) | (0.753-0.840) |
|  |  |  |  |  |  |  |  |
| Cameroon | 1.276 | 1.15 | 0.433*** | 1.047 | 3.935*** | 1.402 | 1.002 |
|  | (0.944-1.725) | (0.862-1.533) | (0.290-0.647) | (0.794-1.380) | (2.326-6.658) | (0.927-2.120) | (0.756-1.327) |
|  |  |  |  |  |  |  |  |
| Cote D'Ivoire | 1.906** | 1.394 | 1.373 | 1.762* | 4.656** | 0.676 | 1.258 |
|  | (1.205-3.015) | (0.886-2.193) | (0.773-2.439) | (1.006-3.085) | (1.750-12.388) | (0.395-1.156) | (0.837-1.890) |
|  |  |  |  |  |  |  |  |
| Eswatini | 1.482*** | 1.126 | 1.048 | 0.807* | 2.941*** | 1.604*** | 0.813* |
|  | (1.242-1.769) | (0.923-1.373) | (0.852-1.288) | (0.678-0.962) | (2.278-3.798) | (1.338-1.923) | (0.682-0.968) |
|  |  |  |  |  |  |  |  |
| Ethiopia | 1.215 |  | 0.822 | 1.105 | 2.035* | 1.35 | 1.186 |
|  | (0.807-1.829) | (NA) | (0.519-1.301) | (0.749-1.631) | (1.052-3.936) | (0.714-2.552) | (0.829-1.696) |
|  |  |  |  |  |  |  |  |
| Kenya | 1.318* | 1.197 | 0.828 | 0.81 | 2.789*** | 1.333 | 0.938 |
|  | (1.019-1.705) | (0.945-1.517) | (0.632-1.085) | (0.476-1.377) | (1.814-4.288) | (0.931-1.911) | (0.743-1.184) |
|  |  |  |  |  |  |  |  |
| Lesotho | 1.399*** | 1.151 | 0.825 | 1.052 | 2.493*** | 1.287* | 0.77** |
|  | (1.189-1.645) | (0.968-1.369) | (0.667-1.020) | (0.892-1.242) | (1.940-3.203) | (1.048-1.581) | (0.657-0.904) |
|  |  |  |  |  |  |  |  |
| Malawi | 1.595*** | 1.304** | 0.885 | 0.976 | 3.065*** | 1.334 | 0.771** |
|  | (1.314-1.938) | (1.072-1.587) | (0.655-1.195) | (0.795-1.198) | (2.247-4.180) | (0.930-1.914) | (0.636-0.935) |
|  |  |  |  |  |  |  |  |
| Namibia | 1.797*** | 1.282* | 1.002 | 0.768** | 2.44*** | 0.977 | 0.672*** |
|  | (1.464-2.204) | (1.028-1.598) | (0.789-1.272) | (0.629-0.938) | (1.810-3.290) | (0.799-1.195) | (0.546-0.828) |
|  |  |  |  |  |  |  |  |
| Rwanda | 1.653** | 0.651* | 1.186 | 1.339 | 2** | 1.983*** | 0.621** |
|  | (1.200-2.276) | (0.462-0.918) | (0.762-1.846) | (0.885-2.027) | (1.222-3.273) | (1.320-2.980) | (0.454-0.849) |
|  |  |  |  |  |  |  |  |
| Tanzania | 1.684*** | 0.883 | 0.675** | 1.098 | 1.957*** | 1.326 | 0.779* |
|  | (1.362-2.083) | (0.717-1.087) | (0.518-0.880) | (0.789-1.529) | (1.375-2.787) | (0.929-1.892) | (0.641-0.946) |
|  |  |  |  |  |  |  |  |
| Uganda | 1.765*** | 0.935 | 0.832 | 0.821 | 3.453*** | 1.076 | 1.131 |
|  | (1.427-2.182) | (0.755-1.159) | (0.645-1.075) | (0.657-1.024) | (2.507-4.756) | (0.727-1.594) | (0.925-1.384) |
|  |  |  |  |  |  |  |  |
| Zambia | 1.271* | 0.82* | 0.646** | 1.179 | 3.567*** | 1.762*** | 0.761** |
|  | (1.053-1.534) | (0.680-0.989) | (0.480-0.872) | (0.986-1.410) | (2.638-4.822) | (1.341-2.316) | (0.635-0.912) |

Notes: VLS=viral load suppression; Confidence interval in parentheses. * p<0.1, ** p<0.05, *** p<0.01.

**Table A8. Blinder-Oaxaca Decomposition by Country for Awareness Outcomes- For Countries with 2 waves of PHIA Survey**

| Country | PHIA Survey | Male | Female | Difference | Endowments | Coefficients | Interaction | n |
| --- | --- | --- | --- | --- | --- | --- | --- | --- |
| Pooled | PHIA 1 | .728*** | .806*** | -.0774*** | .0233*** | -.104*** | .00372 | 17,844 |
|  | PHIA 2 | .867*** | .905*** | -.0380*** | .0118*** | -.0591*** | .00940*** | 17,443 |
|  |  |  |  |  |  |  |  |  |
| Eswatini | PHIA 1 | .823*** | .913*** | -.090*** | .017*** | -.110*** | .004 | 2,981 |
|  | PHIA 2 | .921*** | .954*** | -.0329*** | .0113*** | -.0551*** | .0110* | 2,877 |
|  |  |  |  |  |  |  |  |  |
| Lesotho | PHIA 1 | .773*** | .850*** | -.077*** | .015*** | -.097*** | .005 | 3,188 |
|  | PHIA 2 | .894*** | .917*** | -.0228** | .00690** | -.0389*** | .00924 | 3,638 |
|  |  |  |  |  |  |  |  |  |
| Malawi | PHIA 1 | .716*** | .804*** | -.088*** | .020*** | -.126*** | .018 | 2,215 |
|  | PHIA 2 | .861*** | .913*** | -.0518*** | .0115*** | -.0851*** | .0219** | 2,454 |
|  |  |  |  |  |  |  |  |  |
| Tanzania | PHIA 1 | .538*** | .654*** | -.116*** | .002 | -.133*** | .015 | 1,780 |
|  | PHIA 2 | .829*** | .868*** | -.0392** | .00887* | -.0533** | .00530 | 1,844 |
|  |  |  |  |  |  |  |  |  |
| Uganda | PHIA 1 | .681*** | .764*** | -.083*** | .046*** | -.157*** | .027 | 1,760 |
|  | PHIA 2 | .783*** | .843*** | -.0596*** | .0238*** | -.0844*** | .000934 | 1,478 |
|  |  |  |  |  |  |  |  |  |
| Zambia | PHIA 1 | .698*** | .741*** | -.043** | .026*** | -.081*** | .012 | 2,418 |
|  | PHIA 2 | .857*** | .896*** | -.0386** | .0202*** | -.0568*** | -.00188 | 2,201 |
|  |  |  |  |  |  |  |  |  |
| Zimbabwe | PHIA 1 | .751*** | .820*** | -.069*** | .018*** | -.072*** | -.016 | 3,502 |
|  | PHIA 2 | .858*** | .896*** | -.0380*** | .00360 | -.0649*** | .0233** | 2,951 |

Note: * p<0.1, ** p<0.05, *** p<0.01.

**Table A9. Blinder-Oaxaca Decomposition by Country for Treatment Outcomes- For Countries with 2 waves of PHIA Survey**

| Country | PHIA Survey | Male | Female | Difference | Endowments | Coefficients | Interaction | n |
| --- | --- | --- | --- | --- | --- | --- | --- | --- |
| Pooled | PHIA 1 | .654*** | .730*** | -.0762*** | .0248*** | -.112*** | .0105** | 17,844 |
|  | PHIA 2 | .837*** | .886*** | -.0491*** | .0142*** | -.0730*** | .00967*** | 17,443 |
|  |  |  |  |  |  |  |  |  |
| Eswatini | PHIA 1 | .749*** | .810*** | -.061*** | .020*** | -.085*** | .003 | 2,981 |
|  | PHIA 2 | .885*** | .937*** | -.0520*** | .0147*** | -.0787*** | .0120* | 2,877 |
|  |  |  |  |  |  |  |  |  |
| Lesotho | PHIA 1 | .710*** | .786*** | -.076*** | .014** | -.109*** | .019 | 3,188 |
|  | PHIA 2 | .860*** | .895*** | -.0350*** | .0106*** | -.0581*** | .0125 | 3,638 |
|  |  |  |  |  |  |  |  |  |
| Malawi | PHIA 1 | .638*** | .738*** | -.100*** | .019** | -.151*** | .031* | 2,215 |
|  | PHIA 2 | .843*** | .897*** | -.0541*** | .0152*** | -.0825*** | .0132 | 2,454 |
|  |  |  |  |  |  |  |  |  |
| Tanzania | PHIA 1 | .484*** | .617*** | -.134*** | .001 | -.144*** | .010 | 1,780 |
|  | PHIA 2 | .809*** | .854*** | -.0453** | .0107** | -.0637*** | .00769 | 1,844 |
|  |  |  |  |  |  |  |  |  |
| Uganda | PHIA 1 | .595*** | .704*** | -.109*** | .043*** | -.175*** | .022 | 1,760 |
|  | PHIA 2 | .738*** | .816*** | -.0780*** | .0260*** | -.109*** | .00514 | 1,478 |
|  |  |  |  |  |  |  |  |  |
| Zambia | PHIA 1 | .612*** | .648*** | -.036* | .027** | -.092*** | .030* | 2,418 |
|  | PHIA 2 | .834*** | .879*** | -.0447*** | .0213*** | -.0683*** | .00230 | 2,201 |
|  |  |  |  |  |  |  |  |  |
| Zimbabwe | PHIA 1 | .671*** | .734*** | -.063*** | .029*** | -.077*** | -.015 | 3,502 |
|  | PHIA 2 | .826*** | .876*** | -.0501*** | .00504 | -.0771*** | .0220** | 2,951 |

Note: * p<0.1, ** p<0.05, *** p<0.01.

**Table A10. Blinder-Oaxaca Decomposition by Country for VLS Outcomes- For Countries with 2 waves of PHIA Survey**

| Country | PHIA Survey | Male | Female | Difference | Endowments | Coefficients | Interaction | n |
| --- | --- | --- | --- | --- | --- | --- | --- | --- |
| Pooled | PHIA 1 | .569*** | .649*** | -.0800*** | .0320*** | -.124*** | .0119*** | 17,844 |
|  | PHIA 2 | .782*** | 0.835*** | -.0525*** | .0200*** | -.0832*** | .0107*** | 17,443 |
|  |  |  |  |  |  |  |  |  |
| Eswatini | PHIA 1 | .683*** | .742*** | -.059*** | .028*** | -.106*** | .019* | 2,981 |
|  | PHIA 2 | .855*** | .902*** | -.0470*** | .0231*** | -.0807*** | .0106 | 2,877 |
|  |  |  |  |  |  |  |  |  |
| Lesotho | PHIA 1 | .623*** | .690*** | -.066*** | .018** | .100*** | .015 | 3,188 |
|  | PHIA 2 | .785*** | .826*** | -.0417*** | .0178*** | -.0780*** | .0185** | 3,638 |
|  |  |  |  |  |  |  |  |  |
| Malawi | PHIA 1 | .573*** | .678*** | -.105*** | .029*** | -.149*** | .015 | 2,215 |
|  | PHIA 2 | .814*** | .870*** | -.0557*** | .0169*** | -.0738*** | .00114 | 2,454 |
|  |  |  |  |  |  |  |  |  |
| Tanzania | PHIA 1 | .408*** | .543*** | -.136*** | .007 | -.169*** | .026 | 1,780 |
|  | PHIA 2 | .754*** | .811*** | -.0567*** | .0193*** | -.0785*** | .00242 | 1,844 |
|  |  |  |  |  |  |  |  |  |
| Uganda | PHIA 1 | .481*** | .592*** | -.111*** | .051*** | -.168*** | .006 | 1,760 |
|  | PHIA 2 | .678*** | .756*** | -.0778*** | .0323*** | -.121*** | .0107 | 1,478 |
|  |  |  |  |  |  |  |  |  |
| Zambia | PHIA 1 | .537*** | .584*** | -.047** | .030*** | -.110*** | .033* | 2,418 |
|  | PHIA 2 | .799*** | .841*** | -.0421** | .0275*** | -.0784*** | .00879 | 2,201 |
|  |  |  |  |  |  |  |  |  |
| Zimbabwe | PHIA 1 | .567*** | .645*** | -.077*** | .037*** | -.106*** | -.008 | 3,502 |
|  | PHIA 2 | .741*** | .800*** | -.0590*** | .00696 | -.0869*** | .0209* | 2,951 |

Note: * p<0.1, ** p<0.05, *** p<0.01.
